# The ElectroUteroGraph: A Novel Tool for Assessing Uterine Contractions of Non-Pregnant Women

**DOI:** 10.1109/OJEMB.2022.3159097

**Published:** 2022-03-15

**Authors:** Julius Georgiou, Konstantinos Lekkas, Giorgos Varnava, Marios Sophocleous, Andreas Michaelides, Vasilios Tanos

**Affiliations:** Holistic Electronics Research Laboratory, Department of Electrical and Computer EngineeringUniversity of Cyprus 54557 1678 Nicosia Cyprus; Medical SchoolUniversity of Nicosia, Nicosia, Cyprus and Aretaeio Hospital 121343 2417 Nicosia Cyprus

**Keywords:** Electrode array, electromyography, non-gravid uterus, uterine electrophysiology, uterine peristalsis

## Abstract

*Goal:* Uterine contractility is known to play significant role in women's health. Ultrasonography and magnetic resonance imaging have been used for assessing uterine peristalsis, however they lack practicality, objectivity, and cost-effectiveness. In this paper, the ElectroUteroGraph (EUG) and novel electrodes are introduced, to cover the unmet need of practical intrauterine contractility assessment. The EUG measures biopotentials produced by uterine muscle contraction, similar to the basis of electrocardiography. *Methods*: The EUG was used to fifteen healthy, non-pregnant women of reproductive age. Amplitude and frequency-related features were derived from our recordings. *Results*: The EUG and novel electrodes did not cause any pain or discomfort to the patients, over their multiple recording sessions. The collected data showed difference between the proliferative and luteal phase of menstrual cycle (p < 0.05). *Conclusions*: The EUG can accurately measure uterine electrical activity, in a simple, standardized, safe and pain-free approach, leading to objective evaluation of uterine peristalsis.

## I. Introduction

### A. Background

Uterine peristalsis is one of the key factors for assessing uterine health. Abnormal uterine contractility has been associated with myometrial and sub-endometrial pathologies such us endometriosis, adenomyosis, fibroids and infertility [1], [2]. Particularly for infertility, increased peristaltic activity has been correlated with decreased pregnancy rates [3]–[5]. Considering that 48.5 million couples worldwide are affected by infertility [6], a means to thoroughly investigate uterine contractility is of utmost importance.

In the nongravid uterus, if we exclude highly invasive techniques, such as intrauterine pressure catheters [7], [8], and hysterosalpingoscintigraphy [9], uterine contractility has been measured with ultrasonography (US), and magnetic resonance imaging (MRI) [10]. However, US and especially MRI imaging are costly techniques and require highly experienced operators for contractility assessment. Ultrasound operators introduce high variability in the signal acquisition, as they vary the position and pressure of the transvaginal probe. Furthermore, patient size variability introduces further inconsistencies, requiring extensive case-specific processing methods for extracting reliable contractility information. Hence, they are not easily standardizable techniques and may lead to subjective results. Although some progress has been made for the standardization and automation of MRI [11] and US [12] techniques for the Junctional Zone Endometrium (JZE) contractility measurements, there is still a long way to go before these techniques can become part of the standard gynecological examinations.

An alternative technique for assessing uterine peristalsis is by recording the electrical activity of uterine muscles. As in every muscle, uterine contractions generate electrical biopotentials. Hence, uterine electrical activity can provide information regarding uterine peristalsis. Previous attempts have been made for measuring the ex vivo electrical activity in resected [13] and perfused [14], [15] uteri, as also in vivo [16]. For the in vivo study, it is worth mentioning that sedatives were required, especially for the nulliparous women, making the proposed method unsuitable for routine examinations.

A widely used method for non-invasive electrical measurement of gravid uterus using external electrodes is the electrohysterography (EHG) [17]. The feasibility of using EHG for non-pregnant women was recently investigated [18]. Results only showed statistically significant features, between menstruation and the rest of the menstrual cycle. This was expected, given the cramp-like [19] muscle activity during menses, as opposed to the mild contractions [20] observed outside menses. These mild peristaltic movements vary within this period, in order to promote fertilization and embryo implantation [7]. Hence, external electrodes were not able to track these subtle changes. Furthermore, in a recent publication, multimodal analysis [21] combining US and EHG techniques [12], [18], was successfully used for classifying in vitro fertilization (IVF) results. However, EHG information on its own could not show a statistically significant difference between successful and unsuccessful pregnancy.

### B. The ElectroUteroGraph

In this paper, a novel tool for assessing uterine peristalsis, the ElectroUteroGraph (EUG), is presented. The EUG measures intrauterine electrical activity for non-pregnant women, as the electrocardiograph (ECG) measures the heart's electrical activity, to evaluate the heartbeat and to diagnose significant mechanical dysfunctions. Our tool uses an innovative, flexible, low-profile, intrauterine electrode array. During recordings, the electrode array is in direct contact with the endometrium. Hence, in contrast with distant recordings using external electrodes [18], it can derive direct measurements from JZE's electrical activity in multiple sites with excellent signal-to-noise ratio and minimum interference from other bio-signal sources. Compared to previous electrical measurement attempts using multiple intrauterine catheters [16], we use a single electrode array. Furthermore, due to its low profile and flexibility, our electrode array can conduct measurements without inducing pain and discomfort, as inferred in [16]. The low profile and soft structure do not affect the naturally occurring uterine peristalsis. Given the suppleness of the electrode array, a catheter is required for its placement. The placement follows the standard procedure of embryo transfer, during IVF treatment, which is a well-known procedure amongst gynecologists and does not induce pain.

In this paper a detailed technical analysis is presented regarding the EUG device, including the electrode array placement methodology and the recording process. In order to verify the practicality of the EUG device and the novel electrodes, we conduct a simple study on fifteen healthy, parous, non-pregnant women, of reproductive age across different phases of their menstrual cycle. The results of the study provide evidence of the easy applicability and accurate assessment of contractility. Furthermore, this novel study is of scientific interest for the gynecologists.

## II. Materials and Methods

### A. Patients

Fifteen patients were selected based on inclusion criteria related to demographics, medical history and a clinical examination using transvaginal 2D and 3D ultrasound scans for reassuring that the uterine morphology was suitable for the EUG procedure. The physician provided the volunteers with general information about the procedure and the aims of the EUG research program. Before the data collection, patients had ample time to study the materials at home and to ask further questions, before signing the consent documentation. The EUG study and the associated procedures were approved from the Cyprus National Bioethics Committee, registered as “ΕΕΒΚ/ΕΠ/2019/70”, 23/01/2020. Information regarding the demographics of the patients, their health condition, as well as their uterine and ovarian physiology are shown in Table I.

**TABLE I:** Patients’ Demographics, Health Status and Gynecological Characteristics

**Patients’ Demographic Characteristics**			
	**Average (Mean)**	**Min**	**Max**
Age	37.2	28	48
Number of Children	1.9	1	3
BMI	22.4	18	29.8
**Patients' Health Status & Gynecological Characteristics**			
Ovarian Function	Reproductive Stage		
General Health	Healthy		
Uterine Health	Healthy		
Medication	None		
	**Average (Mean)**	**Min**	**Max**
Age of Menarche	11.9	11	15
Menstrual Cycle Duration	-	24	30
Period Duration	-	2	8
Period Regularity	Regular		

#### 1) Inclusion Criteria

Female adults, over 18 years but within reproductive age, experiencing regular menstrual bleeding, with negative pregnancy test, being sexually active/not virgin, having at least one child, without uterine/cervical scars, without current/past uterine or general health pathologies, without using any medication, with vaginal ultrasound scan prior to EUG test and outside menstruation were included in the study.

### B. The EUG Device

**Figure 1.**
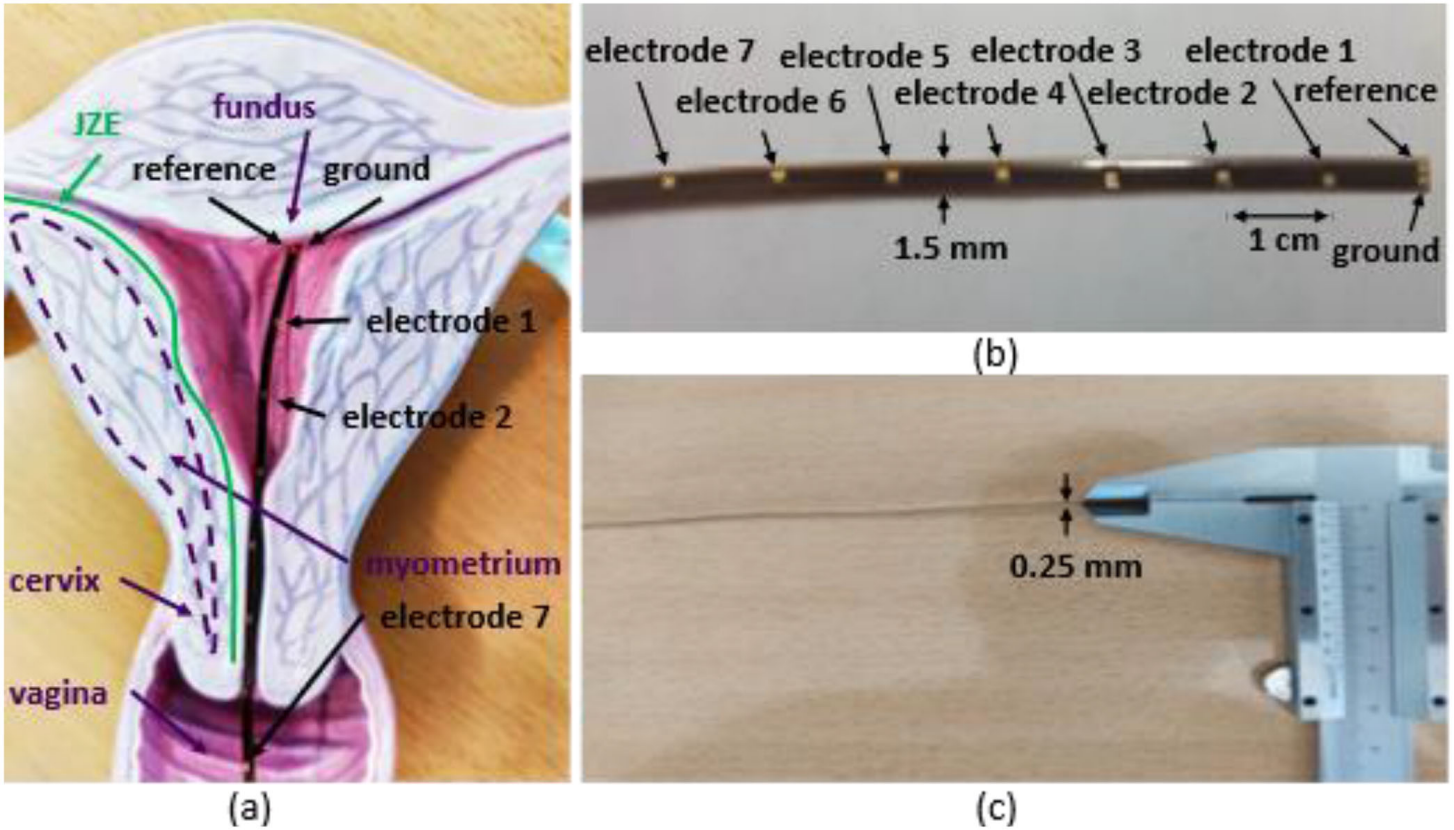
The innovative seven electrode array. (a) The electrode array depicted inside real sized uterine model. (b) Magnified depiction of the used electrode array, with its electrodes labelled. (c) Side view of electrode array.

For the recording of the uterine electrical activity a commercially available multichannel electromyograph (EMG) was used. The device was connected to a disposable, low-profile (400 mm × 1.5 mm × 0.25 mm, length x width x thickness), application-specific, flat, flexible, gold-plated electrode array, that was designed for this purpose by the authors. Note that in a normal uterus, the anterior and posterior uterine walls tend to come into contact with each other, thus providing good electrical contact to the exposed electrode pads as shown in Fig. 1(a); Each electrode array contains seven, jointly encapsulated and insulated, parallel conductors of different lengths, each leading to an exposed electrode. The distributed electrodes record signals from different anatomical locations, running along the centerline of the uterine cavity, with an inter-electrode spacing of 1 cm. At the tip of the electrode array there are two additional electrodes, the reference electrode, and the ground electrode, as shown in Fig. 1(b). The number of electrodes and electrode distance were selected based on the anatomical characteristics of the measured area, so as to provide measuring capability along the entire centerline of the organ, withstanding interpatient anatomical variations. The length of the endometrial cavity is about 3.7 to 4.3 cm for nulli and multi-parous women [22] and the length of the nongravid uterine cervix is about 2.5 cm [23] leading to combined length of less than 7 cm. Hence the electrode length is sufficient for the majority of non-pregnant uteri. The side view of the electrode array is shown in Fig. 1(c), illustrating the low profile needed for recording naturally occurring electrical activity. The electrodes were evaluated for cell toxicity using cell cultures and were shown not to affect cell growth after 1-hour exposure. Note that patient exposure is typically less than 15 minutes. Additionally, all electrode arrays did not show any visible chemical change or mechanical damage after usage. The specifications of the used EMG system are shown in Table II. Furthermore, the system performs impedance measurements, in order to assess the quality of contact between the electrodes and the endometrial wall. Given the high moisture content of the uterus no conductive gel was required, as opposed to other biopotential measurements, such as the electroencephalograph (EEG), the ECG or the electrooculograph (EOG) recordings.

**TABLE II:** Specifications of Multichannel Emg Device

**Parameter**		**Value**
Number of channels	7	
Bandwidth	460 Hz per channel	
Maximal input DC offset	± 250 mV	
Equivalent input noise	1 μV_p-p_^*^	
Maximum sampling frequency	4096 Hz per channel	
Selected sampling rate for EUG	256 samples/s	
Analog to digital (A/D) resolution	16 bits	
Common mode rejection ratio (CMRR)	102 dB^**^	
Isolation mode rejection ratio (IMRR)	140 dB	
Differential input impedance	10 GΩ parallel to 35 pF	

^*^(0.1 Hz–100 Hz), R_in_ < 10 KΩ

^**^(0–60 Hz), all inputs shorted to ground

### C. EUG Measurements Procedure

**Figure 2.**
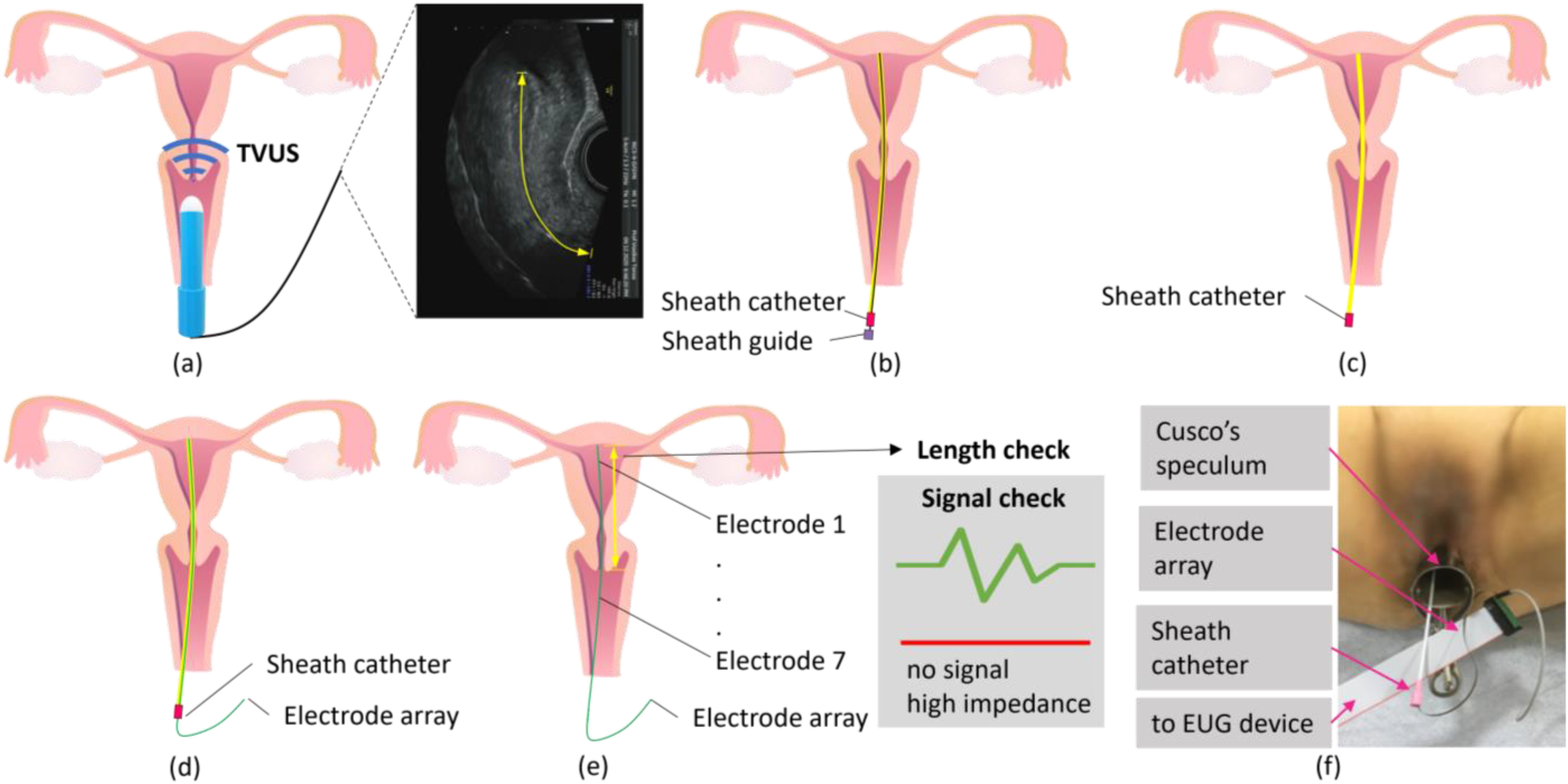
The EUG measurement procedure (Uterine drawing was derived from [28]). (a) Use of TVUS for measurement of fundus to cervix length. (b) Insertion of the sheath catheter with the sheath guide. (c) Removal of the sheath guide. (d) Insertion of the EUG electrode array. (e) Electrodes’ impedance/signal and electrode array's length check. (f) Electrode array in position, with sheath catheter pulled back to expose electrodes to uterine walls.

In each patient the electrode array is placed inside the uterine cavity with the use of a disposable sheath catheter, similar to that used for embryo transfer. Firstly, the patient is placed in lithotomy position and vaginal examination using a Cusco's speculum is performed. Clearance of the cervical os with an antiseptic solution and insertion of a disposable sheath catheter (CooperSurgical Inc., Connecticut, USA) with the sheath guide included, as is done in embryo transfer after IVF (Fig. 2(b)). The sheath guide is a bendable, plastic-coated wire, inserted into the sheath catheter, that assists in the proper placement of the catheter, such that it abuts the fundus. The physician then keeps the catheter in position, whilst the sheath guide is removed (Fig. 2(c)). The gas sterilized EUG electrode array is then inserted into the catheter until it reaches the fundus (Fig. 2(d)). The sheath catheter is then pulled back carefully, whilst maintaining the electrode array in place, in order to expose the electrodes to the uterine walls (Fig. 2(f), in vivo). As the catheter is removed the uterine walls come into contact with the electrode array. The Cusco's speculum is then removed, and the electrode array terminal is connected to the recording device. The electrode placement takes a couple of minutes at most. The electrodes’ position within the endometrial cavity is evaluated by comparing the length of the electrode array inserted in the endometrial cavity with the length of the fundus to cervix, derived by previous transvaginal ultrasound (TVUS) examination (E8 Voluson, GE Healthcare Inc., Illinois, United States) (Fig. 2(a)), as also by checking electrodes’ impedance and active signals (Fig. 2(e)). If some electrodes are outside the cervix, then no signal/high impedance (> 50 kΩ) is detected on the recording device monitor. The patients were asked to remain still and not talk during recordings to minimize the introduction of artefacts. The first few EUG recordings were limited to 5-minute segments, as there was no prior information of patient adherence to the procedure. After the first sessions, it became clear that 10-minute segments were feasible, without introducing patient discomfort. As removing the electrode takes a few seconds at most, the entire EUG recording procedure requires a duration of less than 15 minutes.

According to the experimental protocol, approved by the National Bioethics Committee, the patients were to be asked if they felt period like pains or cramping during the procedure. The response of this cohort, recorded by the physician, was that no pain or substantial discomfort was felt. All analysis was made using MATLAB R2020a (MathWorks Inc., Natick, Massachusetts, USA).

### D. EUG Data Selection

The presented EUG study aims to compare the intensity and frequency changes of the endometrial cavity's electrical signals outside menstruation. Specifically, the monthly cycle days are segmented into two distinct phases; The Proliferative Phase (PP), which corresponds to the time interval between the first day of menstruation and the day before ovulation (commonly considered day 14) and the Luteal Phase (LP), which corresponds to the time interval between the day after ovulation and the day before the next menstruation (commonly considered day 28). In this study all PP data were collected in the non-menstruation part of the PP. To simplify our initial examinations, we focused on examining a subset of the collected EUG data, which is that of the electrode closest to fundal region and has 1 cm distance from the reference electrode (Electrode 1, Fig. 1(a), (b)). The region close to Electrode 1 is particularly important for fertility, since it has been shown that the embryo transfer between 1 to 2 cm from fundus lead to significant increase of pregnancy rate [24].

### E. Signal Processing

**Figure 3.**
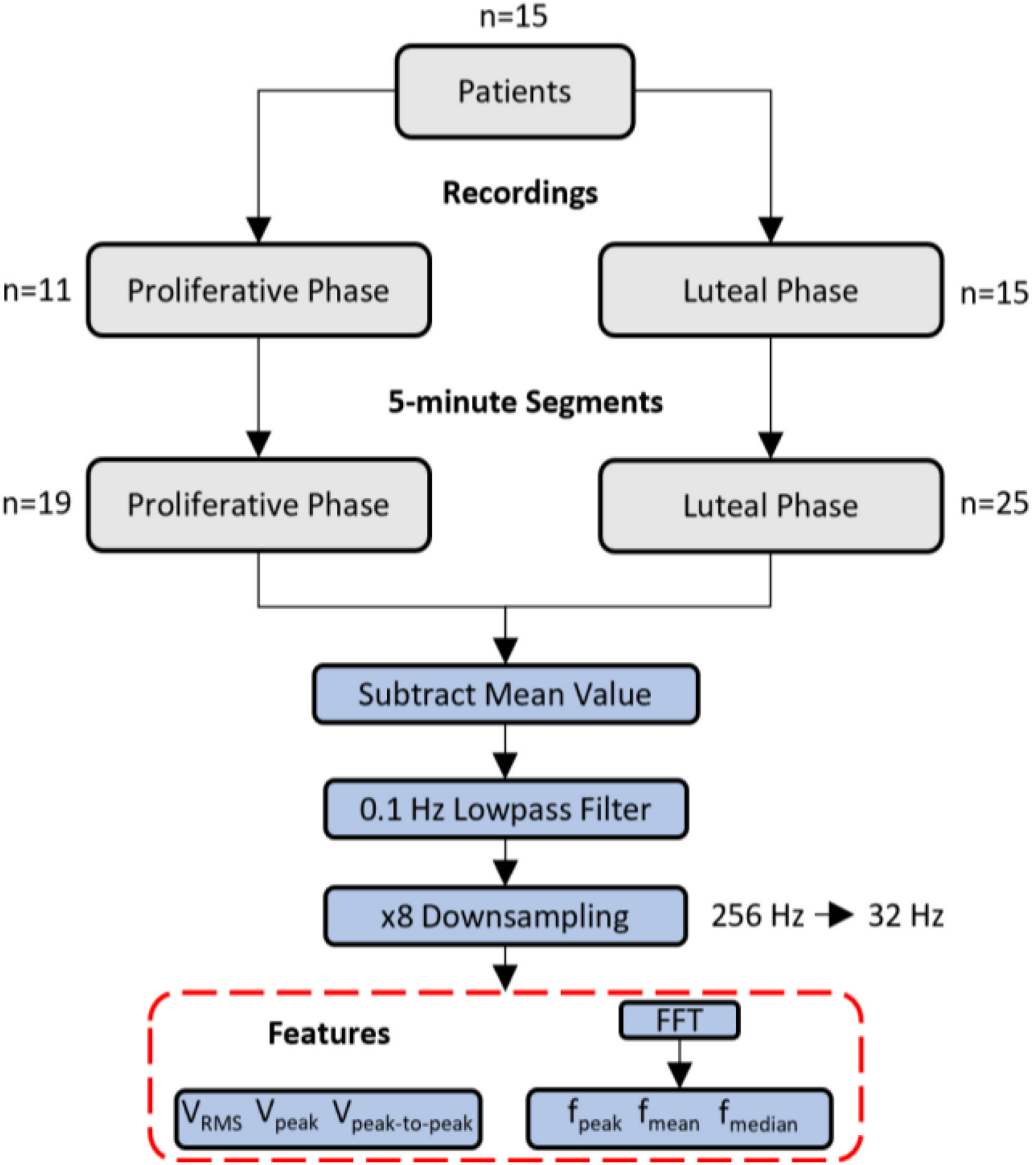
The signal processing procedure that was followed for the extraction of amplitude and frequency-related features. The luteal and proliferative phase recordings were separated into sequential 5-minute intervals. For each 5-minute segment the mean value was subtracted. Next the intervals were filtered with a 0.1 Hz lowpass filter. Next, the segments were downsampled and the features of interest were calculated. A Fast Fourier Transform was applied prior to the calculation of frequency related features.

**Figure 4.**
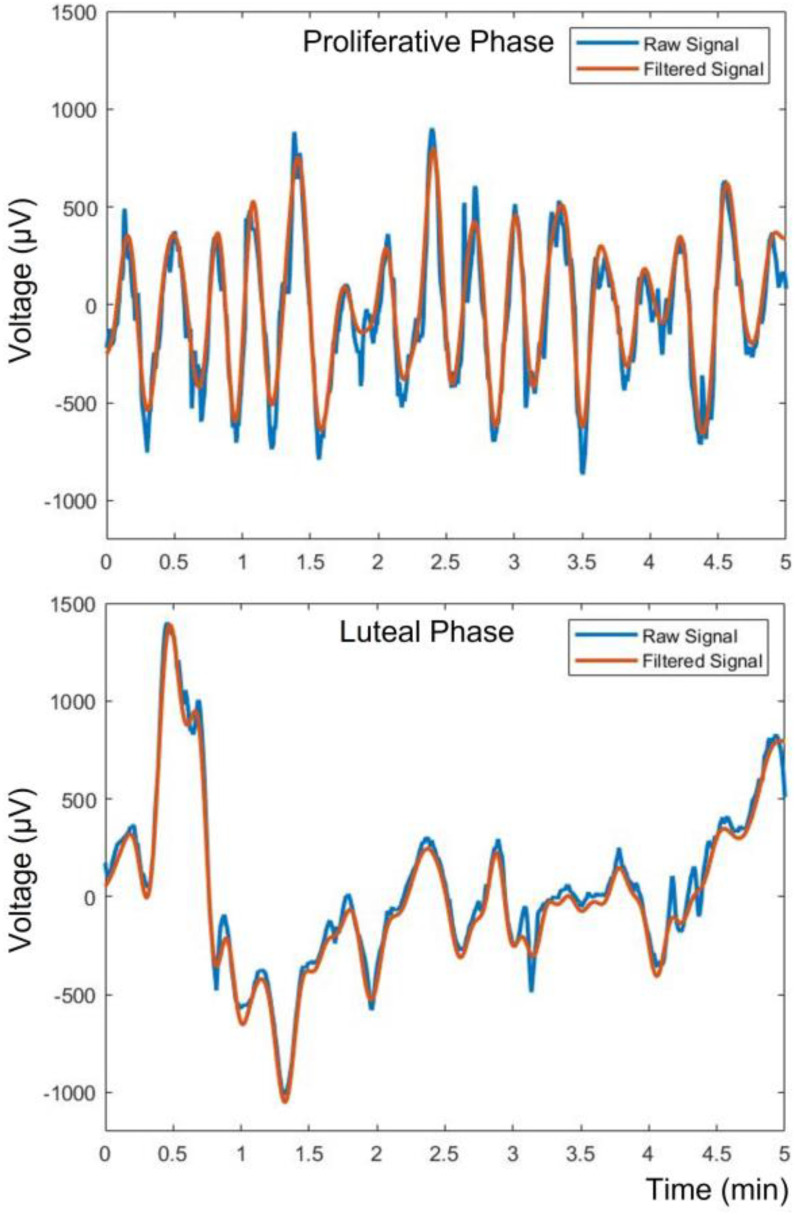
Five-minute segments from monopolar voltage measurements of electrode 1 for PP (top) and LP (bottom), depicting the raw signal (blue) and its filtered version with 0.1 Hz lowpass filter (brown).

In our study, 26 recordings (11 from PP and 15 from LP) were processed. All recordings were divided into sequential 5-minute intervals creating 44 segments (19 from PP and 25 from LP), with maximum of 2 segments per recording. According to prior literature, the maximum expected range of contractions varies between 0.8 and 6 contractions per minute during the whole menstrual cycle for healthy non-pregnant women [25], [26]. The frequency resolution of the Fast Fourier Transform (FFT) for the 5-minute segments (at 32 Hz sampling rate) is 0.0033 Hz, which is equivalent to a lower detection limit of 0.198 contractions per minute. Hence, it is adequate for the application. Regarding the high frequency threshold, a 0.1 Hz lowpass filter was applied for the minimization of high frequency artifacts that are not correlated with the uterine muscles’ activity. Potential artifacts come from 50 Hz noise, the patients’ heartbeat and diaphragm (breathing) biopotentials, setting the upper limit of 6 contractions per minute. For lowpass filtering, an eighth-order Butterworth lowpass filter was applied. The recording device's sampling frequency was 256 Hz. In order to reduce computational time and storage space, after the 0.1 Hz lowpass filtering, which also works as antialiasing filter, waveforms were down-sampled at 32 Hz (x8 down-sampling). Furthermore, because we are only interested in temporal voltage potential changes, so as to track uterine peristaltic activity, the mean value was subtracted from all segments. The aforementioned signal processing procedure is shown in Fig. 3. An example of the electrical activity from two 5-minute segments, in raw format and with their mean value subtracted and filtered with 0.1 Hz lowpass filter, for PP and LP are shown in Fig. 4.

### F. Feature Extraction

For extracting information regarding the intensity as also the frequency characteristics of the intrauterine electrical activity, three amplitude-related and three frequency-related features were selected. The voltage amplitude-related features are the root mean square voltage (*V_RMS_*) (1), the peak voltage (*V_peak_*) (2) and the peak-to-peak voltage (*V_peak__-to-peak_*) (3).

\begin{align*}
&{\mathrm{V}_{\text{RMS}}} = \sqrt {\frac{{\mathop \sum \nolimits_{{\rm{n = 1}}}^{\mathrm{N}} {\mathrm{V}^{2}}\text{[n]}}}{\mathrm{N}}} \tag{1}\\
&{V_{peak}} = max\left({\left| {\mathrm{V}\left[ n \right]} \right|} \right) \tag{2}\\
&{V_{peak - to - peak}} = \max \left({\mathrm{V}\left[ n \right]} \right) - min\left({\mathrm{V}\left[ n \right]} \right) \tag{3}
\end{align*}

where the variable n represents the discrete sample number of the waveform, and ranges between 1 and N, where N is the total number of samples taken in time.

For the frequency-related features, each waveform was transformed to frequency domain using the FFT and the power frequency spectrum was calculated. The frequency-related features are the peak frequency (*f_peak_*) (4), the mean frequency (*f_mean_*) (5) and the median frequency (*f_median_*) (6) of the power spectrum (*P*).

\begin{align*}
{f_{peak}} &= f\left[ {{n_a}} \right]\ :\ P\left({f\left[ {{n_a}} \right]} \right) = \text{max}\left({P\left({f\left[ n \right]} \right)} \right) \tag{4}\\
{f_{mean}} &= \frac{{\mathop \sum \nolimits_{n = 1}^N f\left[ n \right]\ P\left({f\left[ n \right]} \right)}}{{\mathop \sum \nolimits_{n = 1}^N \ P(f\left[ n \right])}} \tag{5}\\
 {f_{median}} &= f\left[ {{n_a}} \right]:\sum\limits_{n = 1}^{{n_a}} {P\left({f\left[ n \right]} \right)} = \sum\limits_{n = {n_a}}^N {P\left({f\left[ n \right]} \right)} = \\ 
&= \frac{1}{2}\sum\limits_{n = 1}^N {P\left({f\left[ n \right]} \right)} \tag{6}
\end{align*}

where the variable n represents the discrete sample number of the waveform, and ranges between 1 and N, where N is the total number of samples taken in time.

### G. Statistical Analysis

For identifying statistically significant difference between the two compared groups, the two-sided Wilcoxon rank sum test was used. P < 0.05 was chosen to show statistical significance. Results are displayed as median value and interquartile range (IQR). Furthermore, because the waveforms were not stationary, the features’ values for each segment were handled as independent samples.

## III. Results

**Figure 5.**
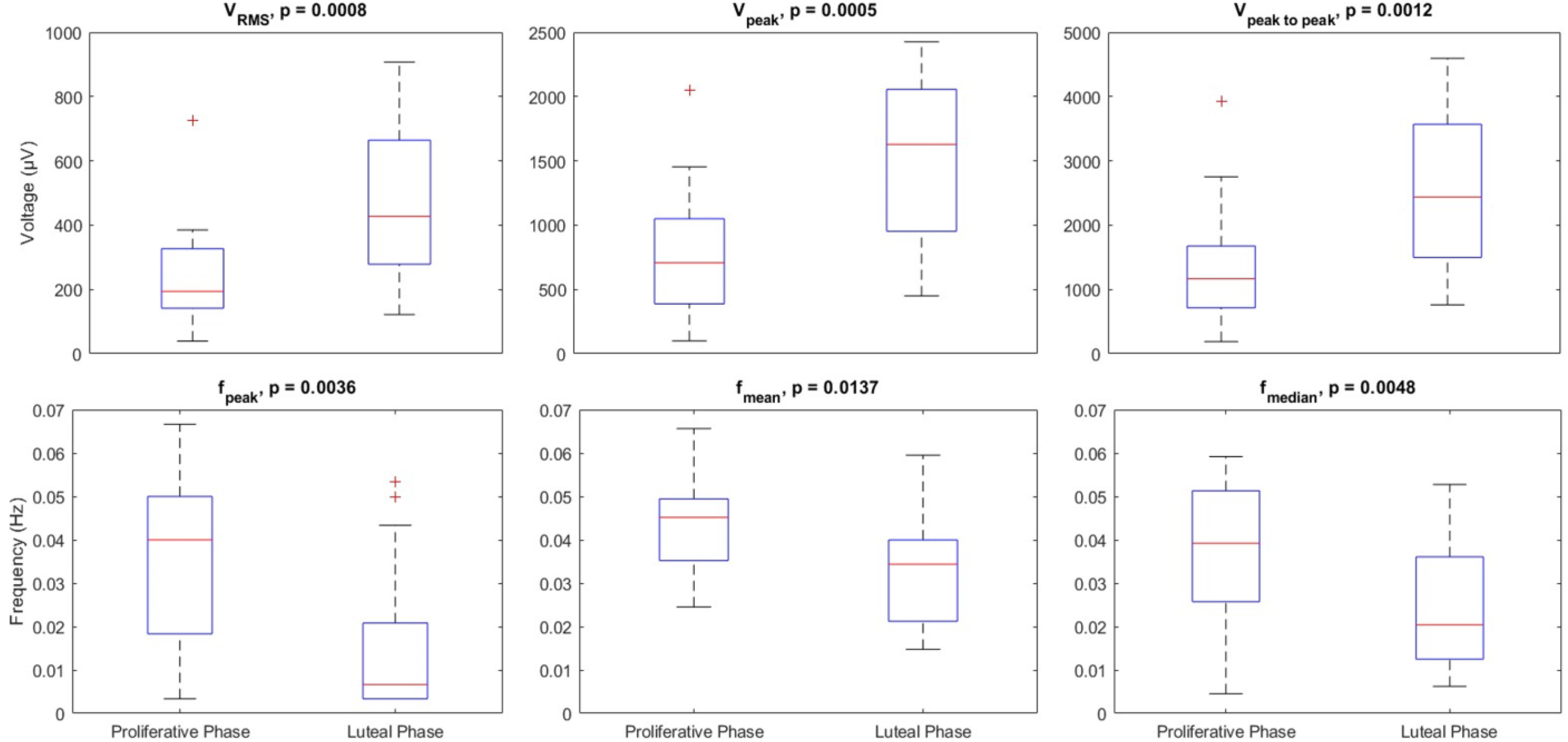
Boxplots for the three extracted voltage amplitude-related features (V_RMS_, *V_peak_*, *V_peak-to-peak_*), and the three extracted frequency-related features (*f_peak_*, *f_mean_*, *f_median_*). P < .05 was selected to show statistically significant difference between groups.

The boxplots comparing the six derived features between PP and LP are shown in (Fig. 5). All amplitude and frequency-related features have shown statistically significant differences between the two selected phases (p < 0.05). For V_RMS_, *V_peak_* and *V_peak-to-peak_* the median values were 193.92 (185.31) μV, 707.38 (663.08) μV and 1167.9 (959.04) μV respectively for PP, compared to 427.57 (385.57) μV, 1628 (1103.1) μV and 2435.1 (2073.7) μV respectively for LP. As it is evident, amplitude-related features showed higher values during LP compared to PP (p < 0.05), revealing that intensity of uterine electrical activity is higher during LP and leading to more intense uterine contractions. For *f_peak_*, *f_mean_* and *f_median_* values were 0.04 (0.0317) Hz, 0.0452 (0.0142) Hz and 0.0392 (0.0256) Hz respectively for PP, compared to 0.0067 (0.0175) Hz, 0.0344 (0.0187) Hz and 0.0204 (0.0236) Hz respectively for LP. As it is obvious, frequency-related features showed higher values during PP compared to LP (p < .05), indicating that frequency of uterine electrical activity is higher during PP and leading to more frequent uterine contractions.

## IV. Discussion

The EHG has been recently used for measuring the electrical activity and indirectly the contractility of non-pregnant uterus [18]. However, there were no statistically significant differences for the compared amplitude and frequency-related EHG derived features among phases of the menstrual cycle, outside menstruation. Non-gravid uterine peristatic movements are created by a small section of the myometrium [10]. Hence, electrical signals might attenuate quickly before reaching external electrodes.

**Figure 6.**
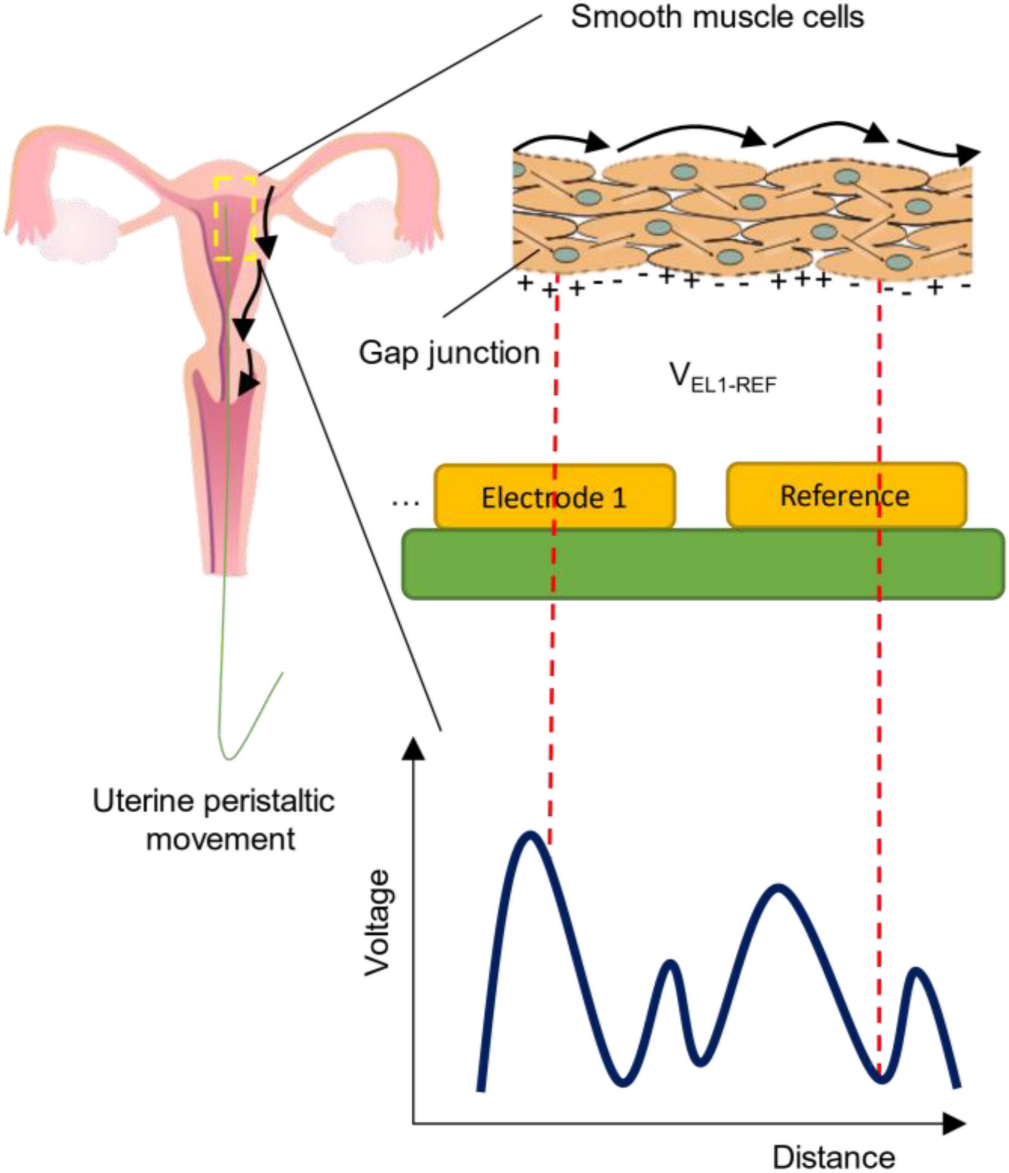
Illustration of the principle behind the measurement of contractions, through voltage potential differences created by the depolarization of smooth muscle cells [29], located in the JZE.

In our study we were able, for the first time, to find in vivo statistically significant differences between different phases of the menstrual cycle, where JZE is the main source of uterine contractility. Furthermore, we observed frequency and intensity variations of JZE's electrical activity, occurring between the PP and LP phases of the menstrual cycle. According to previous literature, uterine contractility, measured via intrauterine pressure variations, has an increasing trend as the menstrual cycle [7] progresses. This agrees with our results, where the amplitude of the electrical activity increases as the menstrual cycle progresses from the PP to the LP part of the cycle. Moreover, the frequency of contractions progressively increases after menstruation, peaking during the periovulatory period. After ovulation and until the end of LP, the frequency of uterine contractions decreases [7], leading to a quiescent state suitable for embryo implantation. Our results, confirm these findings, as would be expected from a physiological standpoint, since muscular contractions are driven by electrical signals of ionic nature. More specifically, the depolarization and repolarization of smooth muscle cells’ membrane, located in the JZE, induces mechanical contractions. With the EUG we were able to track these electrical changes, produced from the ionic movements of muscle activity [27]. A qualitative explanation of the previous points is illustrated in Fig. 6.

Furthermore, in [14] using perfused human uteri, resected during PP and LP, and administering hormones for simulating the hormonal levels, which occur in vivo, PP showed statistically significant higher electrical rhythmic activity compared to LP in fundal region. These results also agree with our findings, providing an additional electrochemical validation to our study.

Previous intrauterine electrodes were much more invasive, requiring sedatives to address the pain [16] and probably induced secondary contractility effects from the applied mechanical stimuli. Hence, normal uterine peristaltic patterns were unlikely to be observed. However, our low-profile (250 μm), flexible, purposely designed, electrode arrays did not create any discomfort or side effects to the patients and did not require the use of sedatives, thus transforming the use of intrauterine electrode arrays for recording JZE's electrical activity into a minimally invasive technique. Furthermore, the proposed technique can be easily implemented from any OB/GYN that has previous experience to IVF with just a demonstration. Additionally, the proposed technique gives consistent results, given that two verification steps for proper electrode placement are part of the application protocol. The EUG costs three orders of magnitude less than an MRI and does not require highly skilled operators and sophisticated signal processing techniques to extract contractility information from US images.

In this initial study for evaluating the novel EUG device, only parous women were selected, since nulli-parous women may have unidentified fertility problems, and hence could add another unknown factor to the study. Prior literature correlates increased contractility with infertility [3], [4]. Furthermore, parous women may be more comfortable or familiar with the procedure. Therefore, for this paper, we defined primi/multi-parity as an extra condition for recruitment.

## V. Conclusion

A simple, objective, and cost-efficient methodology for assessing uterine peristalsis is required to improve on current uterine health evaluation techniques. MRI and US have paved the way for evaluating uterine contractility in a research environment, but have failed to become common practice in OB/GYN and fertility clinics. In this paper we present the EUG, a novel system, along with a standardized methodology, for assessing uterine peristalsis based on recording the electrical activity of endometrial muscles using flexible intrauterine electrode array. The EUG is minimally invasive and OB/GYNs can easily perform the procedure, since the electrode placement is similar to embryo placement during IVF. The EUG was tested on fifteen women, using strict protocols approved by the Cyprus National Bioethics Committee. The post-EUG feedback from the patients indicated that there were no complaints or side effects arising from the EUG recordings. Furthermore, PP recordings showed statistically significant lower amplitude and higher frequency compared to LP. This electrical pattern matches with the peristaltic behavior reported in previous bibliography.

We expect that the EUG will radically expand the toolset that gynecologists have for assessing uterine health. This will materialize once we accurately define healthy EUG activity and compare it to the various uterine pathologies, that interfere with the natural contractility, such us adenomyosis, endometriosis and myomas. It will also open a new chapter in IVF treatment, since it has been proven from multiple studies that excessive uterine contractility negatively affects fertility [3]–[5].
